# Supplementary material for: A highly attenuating and frequency tailorable annular hole phononic crystal for surface acoustic waves
Source: Nat Commun. 2017 Aug 2;8:174. doi: 10.1038/s41467-017-00278-0 (PMC5539253; doi:10.1038/s41467-017-00278-0)
Supplement: Supplementary file 1 — Supplementary Information [file 41467_2017_278_MOESM1_ESM.pdf]

File Name: Supplementary Information
Description: Supplementary Figure and Supplementary Table.

File Name: Peer Review File
Description:

Supplementary Information

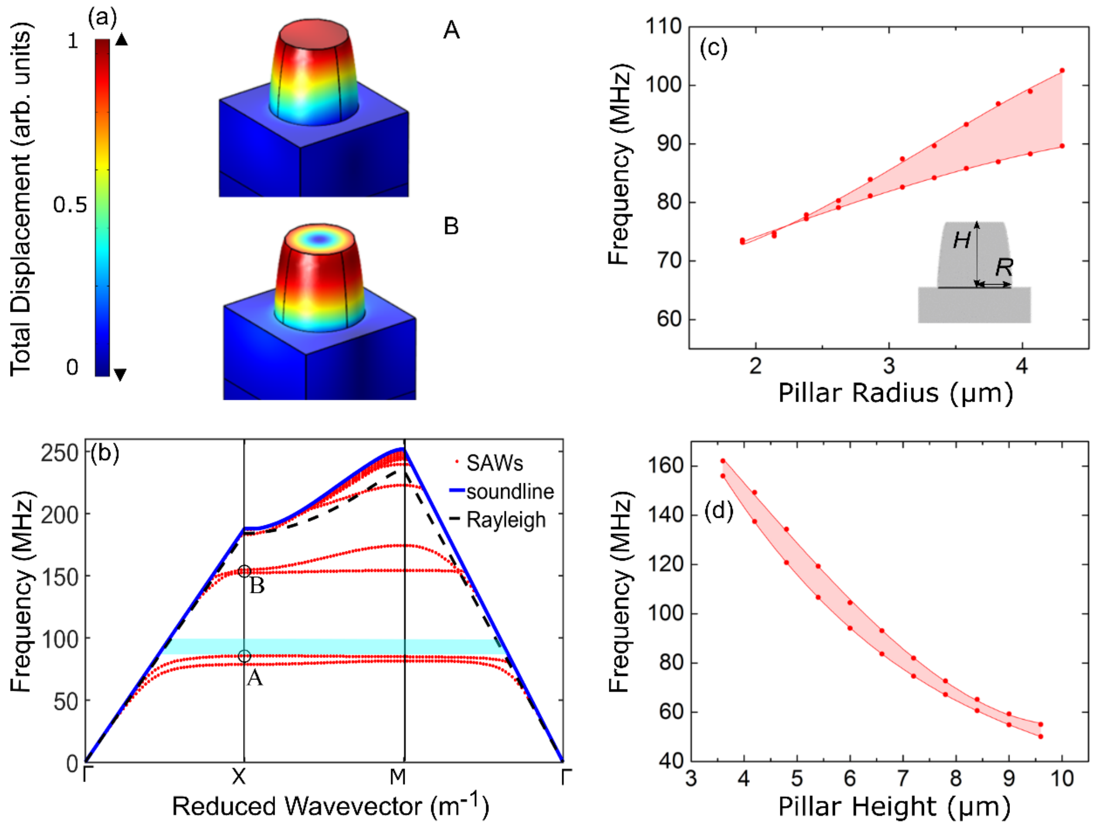

Supplementary Figure 1 | Pillar phononic simulations analogous to annular holes in Figure 1. (a) Total displacement surface plots for modes A and B. **(b)** Surface acoustic wave band diagram generated through finite element method simulation of pillar unit cell with pitch 10.9 µm, radius 4.1 µm and pillar height 6.4 µm. The blue line represents the soundline and the red lines represent propagating surface acoustic wave bands within the 1st Brillouin zone. The teal bar represents the phononic crystal complete bandgap. **(c)** Bandgap limits of pillar phononic crystal against radius of the pillars with cross section schematic of unit cell, with *R* and *H* being the radius, and height of the pillar respectively. The data points represent upper and lower frequency limits of the complete bandgap shaded in red. The lines are polynomial fits to guide. **(d)** Bandgap limits against height of the pillars.

	Attenuation (dB) Hole / Pillar		
Structure Depth/ Height	1 period	9 periods	Frequency (MHz)
5µm	21.4 / 8.9	38.3 / 38.0	120
6.4µm	20.0 / 8.4	26.9 / 22.8	95
9µm	14.4 / 8.1	18.8 / 20.8	57

Supplementary Table 1 | Peak bandgap attenuations. Attenuation versus hole depth / pillar height for 1 and 9 periods of a phononic crystal with associated centre frequencies of bandgaps.
